# BlueBerry Isolate, Pterostilbene, Functions as a Potential Anticancer Stem Cell Agent in Suppressing Irradiation-Mediated Enrichment of Hepatoma Stem Cells

**DOI:** 10.1155/2013/258425

**Published:** 2013-06-26

**Authors:** Chi-Ming Lee, Yen-Hao Su, Thanh-Tuan Huynh, Wei-Hwa Lee, Jeng-Fong Chiou, Yen-Kuang Lin, Michael Hsiao, Chih-Hsiung Wu, Yuh-Feng Lin, Alexander T. H. Wu, Chi-Tai Yeh

**Affiliations:** ^1^Department of Diagnostic Radiology, Taipei Medical University Hospital, Taipei 11031, Taiwan; ^2^Department of Radiology, School of Medicine, College of Medicine, Taipei Medical University, Taipei 11031, Taiwan; ^3^Department of Radiation Oncology, Taipei Medical University Hospital, Taipei 11031, Taiwan; ^4^Departments of Surgery, Taipei Medical University-Shuang Ho Hospital, Taipei 23561, Taiwan; ^5^Graduate Institute of Clinical Medicine, Taipei Medical University, Taipei 11031, Taiwan; ^6^Department of Pathology, Taipei Medical University-Shuang Ho Hospital, Taipei 23561, Taiwan; ^7^Biostatistics and Research Consultation Center, Taipei Medical University, Taipei 11031, Taiwan; ^8^Genomics Research Center, Academia Sinica, Taipei 115, Taiwan; ^9^School of Medical Laboratory Science and Biotechnology, Taipei Medical University, Taipei 11031, Taiwan; ^10^Ph.D. Program for Translational Medicine, College of Medical Science and Technology, Taipei Medical University, Taipei 11031, Taiwan; ^11^Translational Research Laboratory, Cancer Center, Taipei Medical University Hospital, Taipei 11031, Taiwan; ^12^Graduate Institute of Medical Sciences, National Defense Medical Center, Graduate Institute of Medical Sciences, National Defense Medical Center, Taipei 11490, Taiwan

## Abstract

For many malignancies, radiation therapy remains the second option only to surgery in terms of its curative potential. However, radiation-induced tumor cell death is limited by a number of factors, including the adverse response of the tumor microenvironment to the treatment and either intrinsic or acquired mechanisms of evasive resistance, and the existence of cancer stem cells (CSCs). In this study, we demonstrated that using different doses of irradiation led to the enrichment of CD133^+^ Mahlavu cells using flow cytometric method. Subsequently, CD133^+^ Mahlavu cells enriched by irradiation were characterized for their stemness gene expression, self-renewal, migration/invasion abilities, and radiation resistance. Having established irradiation-enriched CD133^+^ Mahlavu cells with CSC properties, we evaluated a phytochemical, pterostilbene (PT), found abundantly in blueberries, against irradiation-enriched CSCs. It was shown that PT treatment dose-dependently reduced the enrichment of CD133^+^ Mahlavu cells upon irradiation; PT treatment also prevented tumor sphere formation, reduced stemness gene expression, and suppressed invasion and migration abilities as well as increasing apoptosis of CD133^+^ Mahlavu CSCs. Based on our experimental data, pterostilbene could be used to prevent the enrichment of CD133^+^ hepatoma CSCs and should be considered for future clinical testing as a combined agent for HCC patients.

## 1. Introduction

Hepatocelluar carcinoma (HCC) represents one of the most common cancer types in the world. The standard treatment options for HCC often involve radiation- and chemo-therapy. Despite advances in the detection and treatment of the disease, mortality rate remains high because current therapies are limited by the emergence of radiation- and chemo-therapy-resistant cancer cells. Existing radiation-therapies against HCC are usually developed against the bulk of the tumor mass, where although they are able to initially shrink the size of the tumor, they fail to eradicate the lesion in full, thus resulting in disease relapse. Recently, HCC progression has been thought to be driven by cancer stem cells (CSC) through their capacity for self-renewal, production of heterogeneous progeny, and resistance to radiation-therapy and to limitlessly divide. The process of re-population has been suggested as the result of accelerated division of stem-cells during treatment (radiation) and/or the enrichment of the CSCs [1]. Therefore, clarification of the radioresistance mechanism is essential for developing novel therapeutic modalities to sensitize hepatoma cells to radiation and improve patient survival.

CSCs are a subpopulation of tumors that are responsible for tumor maintenance and spreading. These cells are characterized to possess unlimited proliferation potential, self-renewal ability, and differentiation capability to generate progenies that constitute the major tumor population [2]. The existence of CSCs has been described in a variety of hematologic and solid tumors including those of the breast, brain, colon, pancreas, lung, liver, and esophagus. CSCs are resistant to many current cancer treatments, including chemo- and radiation therapy [3]. In addition to driving tumorigenesis, CSCs might contribute to distant metastasis and disease relapse [4]. This suggests that the standard interventions, while killing the bulk of tumor cells, may ultimately fail because they do not eliminate CSCs but represent a selection pressure for CSCs.

Since CSCs share similarities with stem cells, stem cell-associated surface markers have been used to identify and isolate CSCs in vitro. For example, leukemia stem cells are enriched in the CD34^+^/CD38^−^ subset of cells and CD133^+^ cells have been implicated as CSCs in many different cancer types including liver [5, 6]. In addition, CSCs can form spherical colonies in suspension cultures characterized and termed tumorspheres. Importantly, isolated CSCs exhibit increased resistance to chemotherapeutic agent and ionizing radiation [2]. Therefore, CSCs have become an important target for drug development.

Pterostilbene (*trans*-3,5-dimethoxy-4′-hydroxystilbene) was first isolated from red sandalwood (*Pterocarpus santalinus*) and subsequently identified in the grape berries of *Vitis vinifera*. Pterostilbene has attracted much attention because it has been demonstrated to have both chemopreventive activity and tumor-killing effects similar to those of resveratrol. For instance, pterostilbene was indicated to induce cell cycle arrest and apoptosis in a variety of cancer cell lines including lung, liver, breast, and pancreas [7]. Recently, it has been reported that pterostilbene prevents azoxymethane- (AOM-) induced colon tumorigenesis in mice via suppressing cancer cell proliferation and the induction of apoptotic pathways [8]. In addition, several pharmacological properties of pterostilbene make it an ideal anticancer agent for development. Structurally, pterostilbene contains two methoxy groups and one hydroxyl group as compared to those of resveratrol which has three hydroxyl groups. The two methoxy groups substantially increase the lipophilicity and oral absorption of pterostilbene leading to a higher potential for cellular uptake. In addition, when administered orally, pterostilbene shows 95% bioavailability while only 20% in the case of resveratrol [9]. Furthermore, pterostilbene's half-life is also seven times longer than resveratrol, 105 min versus 14 min [10].

Collectively, pterostilbene possesses many desired anticancer properties for the development as potential clinical agent. In this study, we evaluated and provided evidence for the use of pterostilbene for targeting and eliminating radiation-enriched CD133^+^ HCC CSCs. 

## 2. Materials and Methods

### 2.1. Materials

Pterostilbene (3,5-dimethoxy-4-hydroxystilbene, 99.7% purity) was generously provided by Professor Chi-Tang Ho (Rutgers University, NJ, USA). MTT dye (tetrazolium dye (thiazolyl blue tetrazolium bromide)) was purchased from Sigma-Aldrich (St. Louis, MO, USA). Primary antibodies c-Myc, CXCR4, COX-2, NF-*κ*B (p65), Twist1, vimentin, E-cadherin, and *β*-actin were purchased from Cell Signaling Technology (Boston, MA, USA). Pterostilbene was dissolved in DMSO and further diluted in sterile culture medium immediately prior to use. A TRIzol RNA isolation kit was obtained from Life Technologies (Rockville, MD, USA); and primers for RT-PCR, dNTP, reverse transcriptase, and Taq polymerase were obtained from Gibco BRL (Cergy Pontoise, France).

### 2.2. Cell Line and Cell Culture

Human hepatoma cell line Mahlavu cells were grown in Dulbecco's modified Eagle's medium (DMEM, Life Technologies, Grand Island, NY, USA) supplemented with 10% (v/v) fetal bovine serum in 5% CO_2_, 37°C humidified incubator. Cell culture and subsequent experiments were used and carried out according to the guidelines established by Environmental and Experimental Safety Committee, Taipei Medical University, Taiwan.

### 2.3. Irradiation

Human HCC cell line, Mahlavu cells were cultured until 80% confluency was reached and subjected to different doses of gamma irradiation (Department of Radiation Oncology, Taipei Medical University Hospital, Taiwan). Briefly, the culture media was replaced with HESS buffer prior to irradiation using a linear accelerator (Varian Medical System, Palo Alto, CA, USA) using 6 megavolts of energy with a source-to-target distance of 100 cm. The cells were placed on a 1 cm bolus and treated with a posterior-anterior direction portal to allow a 1 cm radiation buildup. A radiation absorption doses from 1, 5, and 10 Gy per single fraction were delivered to Mahlavu cells. Surviving cells were subsequently cultured and subjected to flow cytometric analysis. 

### 2.4. Isolation of CD133^+^ Cancer Stem Cells Using Fluorescence Activated Cell Sorting (FACS)

CD133^+^ cells were labeled and sorted using magnetic microbeads (Miltenyi Biotec, Auburn, CA, USA) and labeled with or isotype control antibodies (all from Coulter-Immunotech Co., Miami, FL, USA). Cells were analyzed and sorted on a FACSAria Cell Sorter unit (Becton Dickinson), using propidium iodide (PI) as a viable stain. Cells were gated on low side scatter, low-to-moderate forward scatter, and low PI. For data acquisition, at least 10,000 events were analyzed. The purity and viability of isolated cells were routinely >98%. 

### 2.5. Cell Invasion and Migration Assay

The metastatic ability of CD133^+^ and parental Mahlavu cells was measured using Boyden chamber invasion assay. Briefly, cells were trypsinized, washed with PBS buffer, and resuspended in a serum-free DMEM medium (5 × 10^4^ cells/200 uL) in the presence or absence of pterostilbene. The cells were then seeded into the upper chambers of matrigel coated filter inserts. A serum-containing DMEM medium (500 *μ*L) was added to the lower chambers. After incubating for 24 h at 37°C, filter inserts were removed from the wells, the cells that invaded or migrated through the membrane were stained with propidium iodide and fluorescence images were taken. The invasive cells were then counted using the Analytical Imaging Station software package (Imaging Research, ON, Canada). To measure the migratory ability, cells were seeded into a Boyden chamber with 8 mm pore polycarbonate filters, which were not coated with matrigel. Different concentrations of pterostilbene were used for the evaluation. The migration assay was measured as described in the invasion assay.

### 2.6. Western Blotting

Total cell lysates were prepared using the protein extraction Kit (Panomics, Fremont, CA, USA). Samples (10 mg) of total cell lysates were separated electrophoretically by a 10% SDS-PAGE gel and transferred onto a polyvinylidene fluoride membrane using the BioRad Mini Protean electrotransfer system. The blots were subsequently blocked with 5% skim milk in PBST for 30 min and were probed with primary antibodies, namely, CD133, c-Myc, and COX2 overnight at 4°C. The membranes were sequentially detected with an appropriate peroxidase-conjugated secondary antibody. The blots were washed in PBST, signals were developed using the ECL (enhanced chemiluminescence) detection kit, and images were obtained using UVP BioDoc-It system (Upland, CA, USA).

### 2.7. Statistical Analysis

Statistical Package of Social Sciences software (version 13.0) (SPSS, Inc., Chicago, IL, USA) was used for statistical analysis. Student's *t*-test was used to determine the statistical significance of the differences between experimental groups; *P* values less than 0.05 were considered statistically significant. The level of statistical significance was set at 0.05 for all tests.

## 3. Results

### 3.1. Irradiation-Enriched CD133^+^ Subpopulation of Mahlavu HCC Cells

**Figure 1:**
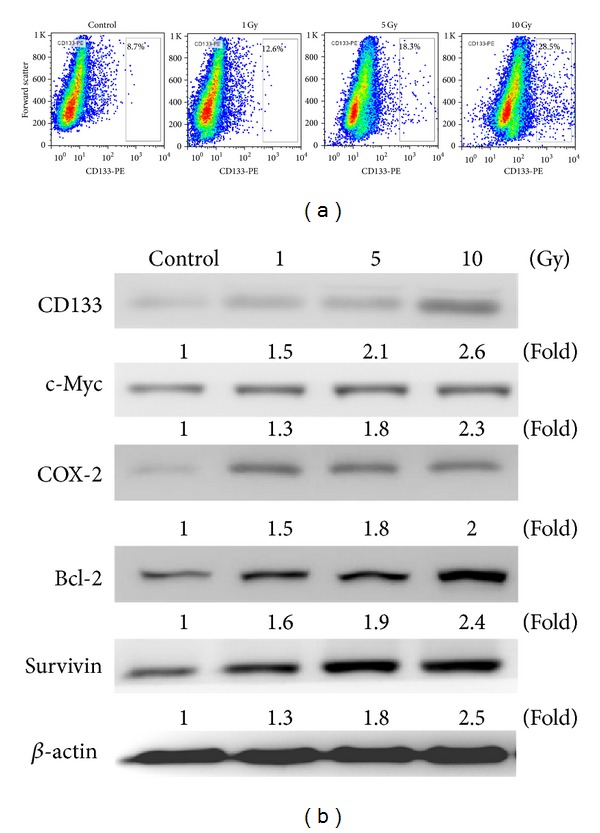
Irradiation increased CD133^+^ subpopulations in HCC cells. (a) Mahlavu cells, subjected to irradiation (1, 5, and 10 Gy), were analyzed using flowcytometric method. (a) Parental Mahlavu cells were found to contain approximately 8.7% CD133^+^ cells, but this subpopulation increased as the dose of irradiation increased that is, 5 Gy to 18.3%. (b) Western blot analysis indicated that CD133^+^ Mahlavu cells were enriched by irradiation and showed an increased expression level of c-Myc (stemness gene) and COX-2 (proinflammatory gene) and Bcl-2 and survivin (prosurvival gene) as compared to their parental counterparts.

Human hepatoma cell line Mahlavu was used as a cell model in this study. Mahlavu cells were irradiated with increasing dose of *γ*-radiation (from 1 to 10 Gy) and subjected to flow cytometric analysis for CD133 expression. We found that the percentage of CD133^+^ subpopulation cells increased as the dose of irradiation increased (Figure 1(a)). This observation supported our hypothesis that irradiation posted a selection pressure for the CD133^+^ subpopulation cancer cells. Subsequently, we characterized the expression profiles between parental and CD133^+^ Mahlavu cells, a significant elevation in the stemness genes including CD133 and c-Myc as well as proinflammatory marker COX-2, and Bcl-2 and survivin (prosurvival gene) were observed (Figure 1(b)). These observations suggested that enrichment of CD133^+^ Mahlavu cells with the characteristics of cancer-stem-like cells. 

### 3.2. CD133^+^ Mahlavu Cells Exhibited Cancer-Stem-Like Cell Properties

**Figure 2:**
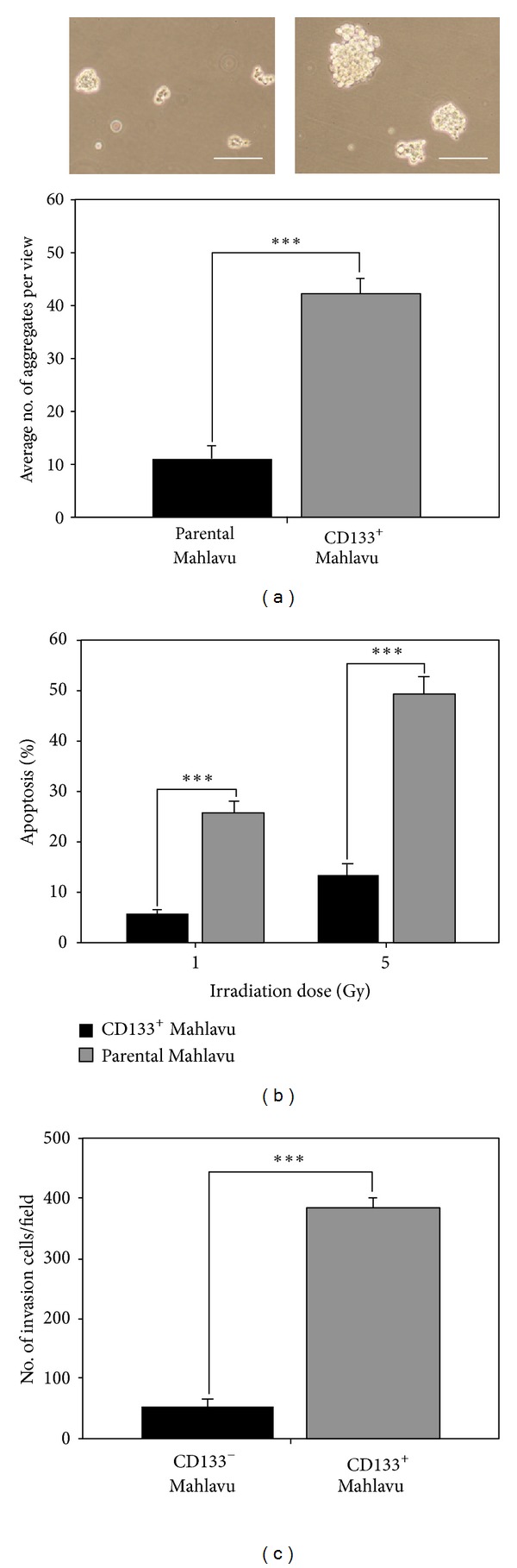
CD133^+^ Mahlavu cells exhibited cancer-stem like cell properties. (a) CD133^+^ Mahlavu cells exhibited a higher ability to form cell aggregates and/or tumor spheroids. (b) CD133^+^ and parental Mahlavu cells were irradiated with 2 different doses (1 and 5 Gy). CD133^+^ Mahlavu cells demonstrated increased resistance against irradiation as compared to CD133-counterparts. (c) CD133^+^ Mahlavu cells were also significantly more invasive than their CD133-counterparts. Bars: 50 *μ*m.

CD133^+^ Mahlavu cells enriched by irradiation were examined for their CSC properties. CD133^+^ Mahlavu cells were found to form a higher number (approximately 4-fold higher) of tumor aggregates and/or spheres as compared to that of parental cells (Figure 2(a)). In addition, CD133^+^ Mahlavu cells were approximately 3 times more resistant towards irradiation comparing to parental Mahlavu counterparts (at 5 Gy, Figure 2(b)). Finally, we also examined the invasive ability of CD133^+^ and parental Mahlavu cells. CD133^+^ Mahlavu cells exhibited an approximately 8-fold increase in their invasive ability when compared to their parental counterparts (Figure 2(c)). Therefore, our data suggested that irradiation could enrich CD133^+^ HCC CSCs characterized by a significantly enhanced malignant properties (as in self-renewal, irradiation-resistant, and invasive abilities). 

### 3.3. Pterostilbene Treatment Suppressed the Percentage of CD133^+^ Mahlavu Cells Enriched by Irradiation and Cancer Stem Cell Properties

**Figure 3:**
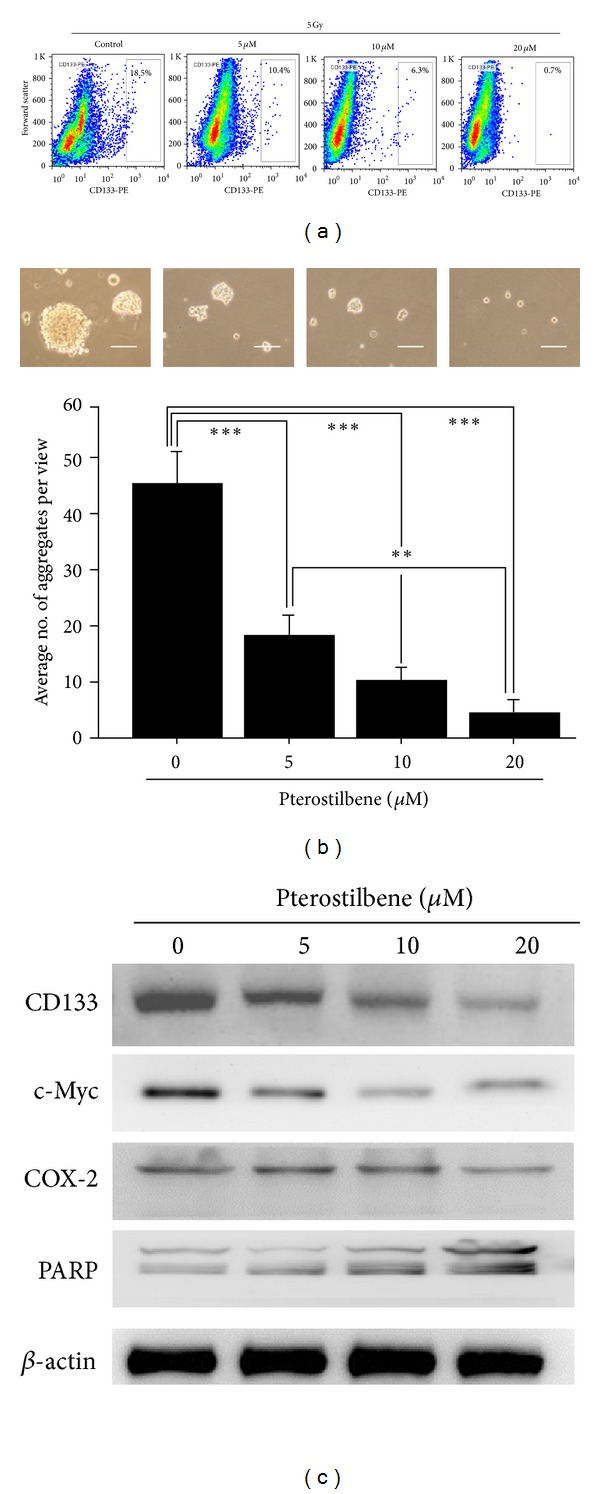
Pterostilbene treatment decreased the percentage of CD133^+^ Mahlavu cells. (a) Irradiated Mahlavu cells were treated with different concentrations of pterostilbene and analyzed for the abundance of CD133^+^ cells. Dose-dependently, pterostilbene decreased the CD133^+^ subpopulation cells. (b) Pterostilbene disrupted the tumor sphere-forming ability of CD133^+^ Mahlavu cells. When pterostilbene was added into the culture medium of CD133^+^ Mahlavu cells, pterostilbene dose-dependently prevented Mahlavu tumor sphere formation. (c) Total protein lysates were obtained from pterostilbene-treated Mahlavu tumor spheres for western blot analysis. The expression levels of stemness markers such as CD133, c-Myc, and proinflammation marker COX-2 were suppressed by the addition of pterostilbene in a dose-dependent manner, while proapoptosis marker PARP was increased under pterostilbene treatment.

Pterostilbene, a stilbenoid chemically related to resveratrol and found in blueberries and grapes, has been implicated for its chemopreventive and anticancer activities. However, the potential anti-CSCs effect of pterostilbene has not been fully examined. In this study, Mahlavu cells were pretreated with an increasing concentrations of pterostilbene (PT, 5, 10 and 20 *μ*M) prior to irradiation. The enrichment of CD133^+^ cells by irradiation (5 Gy) was dose-dependently suppressed by this pretreatment of pterostilbene (Figure 3(a)). For instance, at the highest PT concentration (20 *μ*M), the percentage of CD133^+^ Mahlavu cells was down to approximately 0.7% as compared to 16.5% in the control group. Our data suggested that PT could be specific against CD133^+^ Mahlavu cells so that at high concentrations, CD133^+^ Mahlavu cells were eliminated by its presence. On subsequent observations, pterostilbene dose-dependently suppressed the tumor aggregate/sphere formation ability of CD133^+^ Mahlavu cells (Figure 3(b)). It was evident that the tumor aggregates were significantly disrupted in the presence of PT (insert Figure 3(b)). PT-treated tumor aggregates were harvested for western blot analysis and revealed that PT treatment indeed suppressed stemness gene expression including CD133, c-Myc, and proinflammatory molecule COX-2 (Figure 3(c)). Note that the expression of one of the key proapoptotic molecules, PARP (poly(ADP-ribose) polymerase) was elevated under PT treatment, suggesting that PT also triggered apoptosis in CD133^+^ Mahlavu cells. Furthermore, we have included another cell line SK-Hep-1 to demonstrate that this is not Mahlavu-specific occurrence. Irradiation also enriched CD133^+^ cancer stem-like Sk-Hep-1 HCC cells and pterostilbene treatment suppressed the enrichment of CD133^+^ SK-Hep-1 cells and its stemness (see Supplementary Figures 1 and 2 available online at http://dx.doi.org/10.1155/2013/258425). Taken together, pterostilbene appeared to prevent the generation or enrichment of HCC CSCs by irradiation and suppressed the CSC properties in Mahlavu and SK-Hep-1 cells.

### 3.4. Pterostilbene Treatment Decreased Metastatic Potential in CD133^+^ Mahlavu Cells

**Figure 4:**
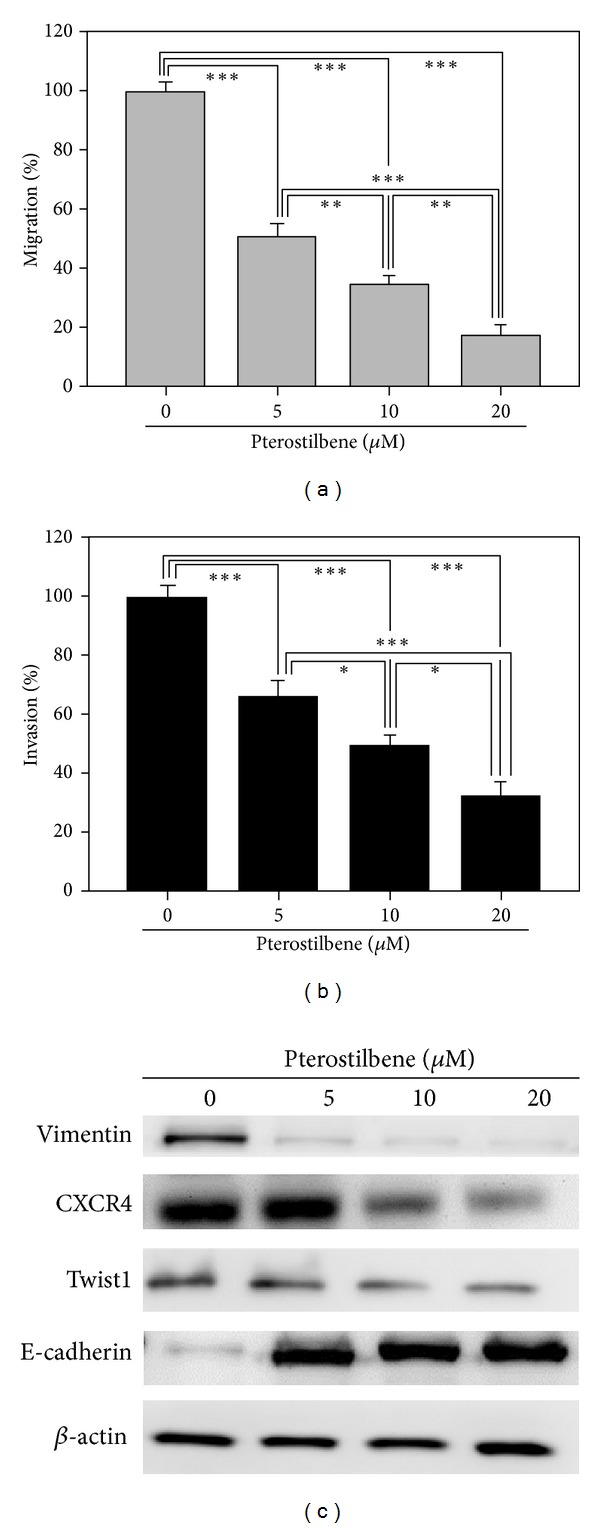
Pterostilbene treatment suppressed the migration and invasion of CD133^+^ Mahlavu cells. (a) CD133^+^ Mahlavu cells were treated with an increasing concentration of pterostilbene (5–15 *μ*M). Pterostilbene treatment led to a dose-dependent suppression in the migration. (b) Similarly, the invasiveness of CD133^+^ Mahlavu cells was decreased as the concentration of pterostilbene increased. (c) Western blot analysis of pterostilbene-treated CD133^+^ Mahlavu cells. Pterostilbene-mediated suppression on migration and invasiveness were correlated to the decreased expression level of prometastasis molecules including vimentin, CXCR4 and Twist1 while the level of epithelial marker E-cadherin increased.

Finally, we evaluated if the addition of PT could affect the metastatic potential of CD133^+^ Mahlavu cells. In the presence of PT (5, 10, and 20 *μ*M), the migration and invasion abilities of CD133^+^ Mahlavu cells were both severely hindered (Figures 4(a) and 4(b), resp.). For instance, at 20 *μ*M, PT reduced the migration ability of CD133^+^ Mahlavu cells down to approximately 20% and invasion ability down to 30% (Figures 4(a) and 4(b), resp.). Mechanistically, PT was found to negatively modulate key molecules associated with metastasis. For instance, the major EMT marker vimentin was reduced in CD133^+^ Mahlavu cells treated with PT in a dose-dependent manner (Figure 4(c)). Mice which received pterostilbene demonstrated significantly lower tumor burden, as evident by gross tumor volume (Supplementary Figure 3). In addition, chemotactic associated molecule CXCR4 was downregulated in the presence of PT. Importantly, one of the key transcription factors associated with epithelial-to-mesenchymal transition (EMT), Twist1, was also downregulated by pterostilbene treatment. 

## 4. Discussion and Conclusions

One of the most challenging tasks in managing liver cancer is the high incidence of treatment resistance. The presence of cancer stem cells was been suggested to play important role in treatment resistance and disease progression. However, as how the CSCs are generated remains poorly understood. Emerging evidence has implicated that current therapeutic strategies may actually help enrich and select for treatment-resistant CSCs [1, 3]. In the present study, we demonstrated that in the wake of irradiation, the surviving Mahlavu cells represented a subpopulation of cells which were not only CD133^+^ but also treatment-resistant. In addition, under serum-deprived culture condition, these CD133^+^ Mahlavu cells demonstrated enhanced ability to form tumor aggregates and/or spheres, a key characteristic of cancer stem cells [4]. Similarly, it has been reported that irradiation increased the number of CD133^+^ glioma stem cells and CD24 (-/low) breast cancer stem cells [11, 12], and these cancer stem cells played a major role in radiation resistance. Taken together, these observations provide an important rationale for developing alternative treatment strategies for HCC management. 

**Figure 5:**
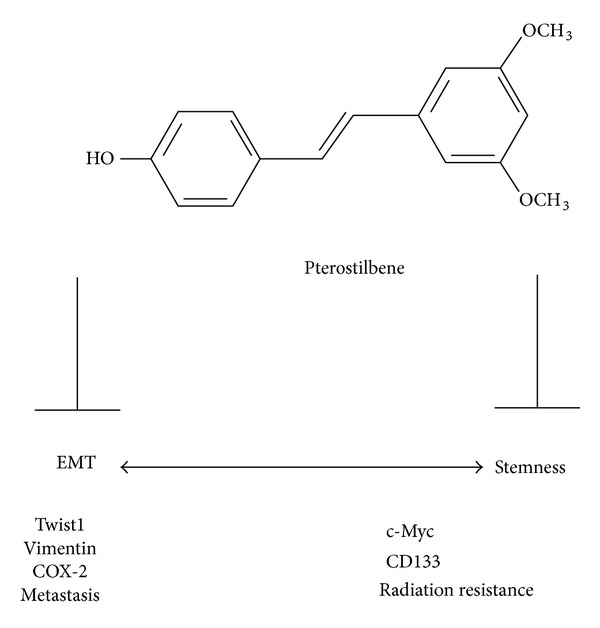
Proposed mechanism for pterostilbene-mediated anti-CSC effects. Pterostilbene suppresses CSC generation by negatively regulating the expression levels of c-Myc and CD133 and inhibiting EMT by downregulating Twist1, vimentin, and COX-2 expressions. The double arrow implies that EMT is associated with the increasing stemness of cancer stem cells. Blunt arrow represents suppression.

The natural occurring pterostilbene (*trans*-3,5-dimethoxy-4-hydroxystilbene) is an antioxidant predominantly found in blueberries, grapes, and tree wood [9, 13]. Pterostilbene and resveratrol have similar pharmacologic properties; however, pterostilbene has several advantages. For instance, pterostilbene contains two methoxy groups and one hydroxyl group whereas resveratrol has three hydroxyl groups. The two methoxy groups have been suggested to substantially increase pterostilbene's lipophilicity and oral absorption, resulting in a higher potential for cellular uptake. In addition, when administered orally, pterostilbene shows 95% bioavailability while resveratrol only has 20% bioavailability [13]. More importantly, Pterostilbene's half-life has been estimated approximately seven times longer than that of resveratrol [10]. Taken together, pterostilbene represents a more superior candidate for anticancer agent over resveratrol. Based on these premises, pterostilbene was selected and evaluated for its potential in targeting and eliminating HCC CSCs. In the present study, we provided in vitro evidence that pterostilbene treatment prevented the enrichment of CD133^+^ Mahlavu cells generated by irradiation and/or promoted apoptosis of these CD133^+^ cells. Importantly, pterostilbene treatment significantly suppressed the cancer stem cell properties of CD133^+^ Mahlavu cells, namely, tumor aggregate/sphere formation as well as inhibited migration/invasion abilities of CD133^+^ Mahlavu cells. Although the precise mechanism as how PT treatment contributed to the prevention of CSC generation by irradiation is not clear, it is likely that PT as a potent antioxidant fended off irradiation-induced oxidative stress and inflammation by suppressing the expression level of COX-2 [7]. Importantly, COX-2 has been indicated as a key molecule in cancer-associated inflammation as well as the promoter of epithelial-to-mesenchymal transition (EMT) in liver cancer [14]. This is in agreement with our observations that PT treatment negatively regulated major EMT markers including CXCR4, vimentin, and Twist1, all of which have been associated with cancer metastasis and poor prognosis [15]. Studies have indicated that the propensity of EMT is associated with the generation of breast CSCs [16, 17]. Thus, we proposed that PT suppressed the generation of irradiation-induced CD133^+^ HCC CSCs by targeting key EMT molecules (Figure 5). 

Notably, PT treatment also reduced the expression of c-Myc, a potent stemness and oncogene [18]. The suppression of c-Myc expression by PT might have a direct impact on several oncogenic properties. First, the disruption of tumor aggregates/spheres in the presence of PT could be the result of PT-mediated c-Myc downregulation, which led to cellular differentiation. Second, the suppression of c-Myc might also contribute to the decreased radiation resistance in CD133^+^ Mahlavu CSCs. It has been shown that the modulation of c-Myc leads to radiation sensitization of hepatoma cells [19, 20]. Furthermore, our unpublished data showed that PT treatment could suppress another major molecule, survivin, which has been implicated for radiation resistance in glioma [21]. 

Interestingly, according to our in vitro data, pterostilbene (at low concentrations ranging from 2.5 to 10 *μ*M) posed inhibitory activity against Mahlavu tumor spheres. In agreement, pterostilbene was also shown to exert potent antiproliferative ability against MCF7 breast cancer cells [22]. In our in vivo study, 5 mg/Kg daily intraperitoneal injection of pterostilbene could significantly delay and suppress the onset of M2 TAM cocultured MDA-MB-231 tumorigenesis [23]. Our in vitro and in vivo data suggests that pterostilbene could reduce the enrichment of CD133^+^ Mahlavu cells upon irradiation and target the liver cancer stem cells. This low concentration of pterostilbene may be achievable in vivo. According to a previous study [24], pterostilbene has an estimated bioavailability of 12.4%, a half-life of 2.38 ± 0.84 h, and a glucuronidated pterostilbene metabolite with half-life of ~8 hours excreted in urine.

In conclusion, our current study demonstrated that irradiation could enrich CD133^+^ subpopulation of Mahlavu cells. These cells possess cancer stem cell characteristics such as increased tumor sphere forming ability, enhanced resistance towards irradiation, and elevated metastatic potential. In the presence of pterostilbene, these aforementioned phenomenea could be suppressed via the downregulation of c-Myc, COX-2, and EMT markers such as vimentin, CXCR4, and Twist1. Therefore, pterostilbene could be used in combination with radiation therapy to prevent the generation of CSCs and possibly leads to an improved outcome in HCC patients.

## Supplementary Material

S1. Tumor xenografts on NOD/SCID mice:
The effects of pterostilbene on the tumorigenicity of CD133+ Mahlavu cells were evaluated on NOD/SCID mice. CD133+ Mahlavu cells (1x10^4^) were pretreated with or without 5 *μ*M of pterostilbene for 24 hrs, and all of the cells were then collected and injected subcutaneously into NOD/SCID mice. Forty days after inoculation, the final tumor size was measured with a caliper (calculated volume = shortest diameter^2^ × longest diameter/2). All animal procedures were approved by the Institutional Animal Care and Use Committee at Taipei Medical University (Approved protocol No. LAC-101-0226). The error bars indicate standard error of the average. Significance was determined by the Student *t*-test. All animals were humanely sacrificed after 4 weeks of monitoring due to the excessive tumor burden.
