# Supplementary material for: BlueBerry Isolate, Pterostilbene, Functions as a Potential Anticancer Stem Cell Agent in Suppressing Irradiation-Mediated Enrichment of Hepatoma Stem Cells
Source: Evid Based Complement Alternat Med. 2013 Jun 26;2013:258425. doi: 10.1155/2013/258425 (PMC3710633; doi:10.1155/2013/258425)
Supplement: Supplementary file 1 — S1. Tumor xenografts on NOD/SCID mice: The effects of pterostilbene on the tumorigenicity of CD133+ Mahlavu cells were evaluated on NOD/SCID mice. CD133+ Mahlavu cells (1x10^4^) were pretreated with or without 5 μM of pterostilbene for 24 hrs, and all of the cells were then collected and injected subcutaneously into NOD/SCID mice. Forty days after inoculation, the final tumor size was measured with a caliper (calculated volume = shortest diameter2 × longest diameter/2). All animal procedures were approved by the Institutional Animal Care and Use Committee at Taipei Medical University (Approved protocol No. LAC-101-0226). The error bars indicate standard error of the average. Significance was determined by the Student t-test. All animals were humanely sacrificed after 4 weeks of monitoring due to the excessive tumor burden. [file 258425.f1.docx]

Supplementary Figure 1.

Supplementary Figure 2.

Supplementary Figure 3.
